# Urosymphyseal fistulas in women: identification and characterization of a previously undescribed phenomenon

**DOI:** 10.1093/jscr/rjab331

**Published:** 2021-08-10

**Authors:** Uzoma A Anele, Hadley M Wood, Kenneth W Angermeier

**Affiliations:** Center for Genitourinary Reconstruction, Department of Urology, Glickman Urological and Kidney Institute, Cleveland Clinic Foundation, Cleveland, OH, USA; Center for Genitourinary Reconstruction, Department of Urology, Glickman Urological and Kidney Institute, Cleveland Clinic Foundation, Cleveland, OH, USA

## Abstract

Urosymphyseal fistula (USF) is a very uncommon but catastrophic condition which typically occurs in the setting of previous radiation treatment for prostate cancer. As a result, USF has only been described in male populations. For the first time, we characterize this phenomenon in a series consisting of four women managed at our quaternary institution. We found that most patients presented with pubic pain and all were diagnosed with USF on CT or MRI. Imaging commonly demonstrated features suggesting osteomyelitis which was confirmed on histology. Patients experienced significant decreases in peri-operative pain scores following extirpative surgery with urinary diversion, bone debridement/resection and tissue interposition. This case series highlights the rarity of USF in women in order to help promote increased recognition and timely management.

## INTRODUCTION

Urosymphyseal fistula (USF) is a very uncommon but devastating condition often occurring after endoscopic bladder outlet procedures (EBOP) performed in the setting of previous radiotherapy for prostate cancer [1]. Consequently, the majority of literature has reported USF in males with a notable absence of descriptive studies in women [1–6]. Therefore, we present the first characterization of this phenomenon in a series of women at our quaternary care institution.

## CASE SERIES

### Case selection and patient management

We performed a single-center retrospective study identifying women among all adult patients with USFs treated between January 2009 and December 2020. Patients were diagnosed primarily based on clinical presentation and imaging. Definitive management was extirpative, involving cystectomy with ileal conduit urinary diversion, pubic bone debridement/resection and tissue pedicle flap. Treatment typically required multidisciplinary coordination between reconstructive urology, orthopedic surgery, plastic surgery and infectious disease (ID) specialists.

#### Case 1

She is a 79-year-old woman with a history of chemoradiation 29 years ago for anal cancer who presented with month-long bilateral groin pain and recurrent urinary tract infections. She previously underwent urethral bulking with endoscopic collagen injection for stress urinary incontinence 7 years ago. MRI was performed and concerning for possible colovesical fistula with pubosymphyseal involvement. Exam under anesthesia with cystoscopy and proctosigmoidoscopy failed to demonstrate clear evidence of fistulization between bowel and bladder. CT cystogram was performed and demonstrated an anterior bladder wall defect communicating along the midline with the lower abdominal wall, as well as pubosymphyseal osteomyelitis. After surgical intervention, she was discharged on intravenous vancomycin, fluconazole and flagyl per ID. One year later, she developed severe vaginal and urethral pain. She was found to have a urethrocutaneous fistula and abscess requiring urethrectomy and drainage via a transvaginal approach.

**Table 1:** Demographics and clinical features

Case	Age (years)	BMI (kg/m^2^)	Medical comorbidities	Prior pelvic radiation and time to diagnosis (years)	Prior EBOP or catheterization	Time from prior EBOP or catheterization to diagnosis (months)	Time from prior EBOP or catheterization to treatment (months)
1	79	24.0	Htn, DM, CKD, CAD, MI, Anal Ca	Yes, 28.9	Urethral bulking	79	82
2	61	36.8	Htn, DM, AIH, Lupus, MVD, OSA, Paraplegia	No	Chronic indwelling urethral catheter	NA	NA
3	66	34.4	Htn, DM, MS, OSA	No	Chronic indwelling urethral catheter	NA	NA
4	66	25.9	Htn, DVT, Endo Ca	Yes, 1.9	No	NA	NA

Htn = Hypertension, DM = Diabetes Mellitus, CKD = Chronic Kidney Disease, CAD = Coronary Artery Disease, MI = Myocardial Infarction, Anal Ca = Anal Cancer, AIH = Autoimmune Hepatitis, MVD = Mitral Valve Disease, OSA = Obstructive Sleep Apnea, MS = Multiple Sclerosis, DVT = Deep Vein Thrombosis, Endo Ca = Endometrial Cancer, NA = Not Applicable

#### Case 2

She is a 61-year-old woman with a history of paraplegia consequent to an epidural abscess and burst fracture 1 year prior, and neurogenic bladder managed with a chronically indwelling Foley catheter who presented with urethral/vaginal discharge and pubic pain. She was transferred to our institution after diagnosis of pubic osteomyelitis on CT. Subsequent CT cystogram revealed a fistula arising from the mid-distal urethra with pubosymphyseal extension involving erosive changes and gas foci. She clinically improved on intravenous meropenem and then underwent examination under anesthesia with cystoscopy and open suprapubic catheter placement. Following a 3-month period of clinical optimization, she was admitted for pre-operative intravenous meropenem and extirpative surgery. She recovered well postoperatively and was discharged on intravenous linezolid, ertapenem and fluconazole per ID. She was readmitted briefly approximately 1 month later for resuscitation from dehydration.

#### Case 3

She is a 66-year-old woman with a history of multiple sclerosis and neurogenic bladder managed for over 1 year with a chronically indwelling urethral Foley catheter. She was referred to the emergency department for leukocytosis and abnormal CT (assessing abdominal pain and decubitus ulcer) by her generalist. Imaging demonstrated a septated fluid collection in the left groin with pubosymphyseal and left pubis involvement which elicited concern for acute osteomyelitis (later confirmed on MRI). Clinical exam demonstrated a patulous urethral meatus draining pus and an anterior urethral defect with palpable bone. She underwent surgical intervention including colostomy for fecal diversion in managing a stage-4 sacral decubitus ulcer. Post-operatively, she developed a puboperineal abscess requiring surgical incision and drainage 7 days later. She was ultimately discharged on intravenous daptomycin, ciprofloxacin and flagyl per ID. Stomal revision and parastomal hernia repair were performed 7 months later. She passed away the following year from undisclosed causes.

#### Case 4

She is a 66-year-old woman with a history of endometrial cancer and chemoradiotherapy following hysterectomy. She sustained a pelvic fracture 11 months afterwards with initially suspected disease recurrence on MRI prompting eventual PET/CT and multiple biopsies which were negative. She continued to have pain and gait issues for another year and developed a right thigh abscess confirmed by CT. Additionally, a possible fistulous communication with the bladder and left pelvis was noted; however, this was not further evaluated or definitively managed beyond abscess drainage at that time. Repeat CT 1 month later was notable for pubosymphyseal diastasis with fragmented pubic bone fractures. Approximately 2 years later, she developed sepsis due to a right groin abscess which was drained by general surgery; however, this re-accumulated 1 week later prompting percutaneous drainage. Upon noting high drain output with low urinary output, an elevated drain creatinine level was determined and raised concern for a urinary fistula. CT cystogram subsequently confirmed the fistula and her care was transferred to our institution after receiving bilateral nephrostomy tube and urethral catheter diversions. Three months later, she underwent surgical extirpation with rectus muscle interposition flap. Post-operatively, she developed an ileus requiring 3-day nasogastric decompression. She was discharged on ceftriaxone.

## DISCUSSION

**Figure 1:**
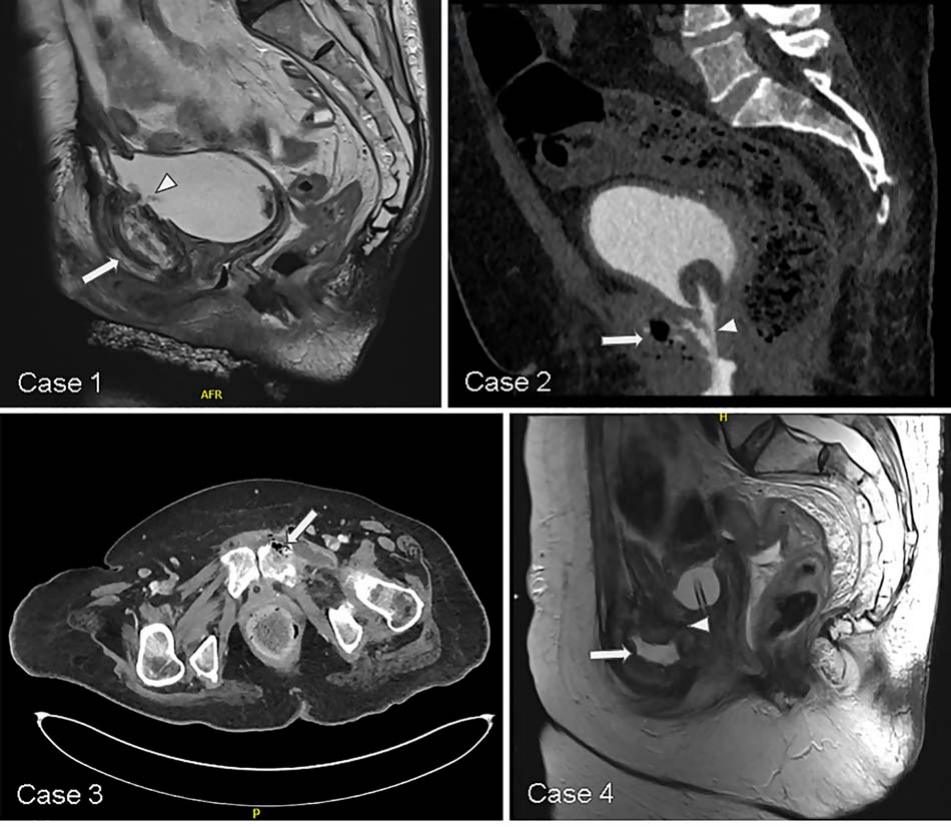
Representative CT and MRI imaging demonstrating USF (white arrowhead) and features suggesting pubic osteomyelitis (white arrow) in each respective case.

We identified four women, median age 66 (IQR 4.5) years old, who underwent USF management within a narrower period of August 2017 and May 2019 (Table 1). Three patients presented with pubic pain and all were diagnosed with USF on CT or MRI (Table 2). Pubic edema, enhancement and erosion were common features suggesting osteomyelitis associated with the fistulas (Fig. 1). Patients ultimately underwent extirpative surgery with urinary diversion, bone debridement/resection and tissue interposition. All were confirmed to have pubic osteomyelitis on histology (Table 2).

**Table 2:** Perioperative characteristics and outcomes

Case	Modality for initial USF diagnosis	Pre-operative urine culture organism species	Intra-operative tissue/bone culture organism species	Pre-operative imaging suggesting pubic osteomyelitis	Histology demonstrating osteomyelitis	Pain score Preop|Postop	Chronic opioid use Preop|Postop	Post-operative complication, Clavien-Dindo grade	Follow-up (months)
1	MRI	Enterococcus, Candida	Enterococcus, Candida	Yes	Yes	8|NR	Yes|No	No	24
2	CT	*Escherichia coli*, Proteus, Pseudomonas	Citrobacter, Morganella, Enterococcus, Proteus, Candida	Yes	Yes	0|0	No|No	No	37
3	CT	No growth	Enterococcus	Yes	Yes	10|0	No|No	Yes, 3b	14
4	CT	No growth	No growth	No	Yes	7|4	Yes|Yes	Yes, 1	17

NR = Not Recorded

USF is an uncommon condition typically observed in men in the context of prior prostate cancer radiotherapy and often following EBOP [1]. This condition is progressively destructive, leading to pubic osteomyelitis and periodically local abscess formation [1, 5]. Thus, patients may present with pubic pain and experience delayed diagnosis due to lapses in timely recognition. Definitive management in most cases warrants cystectomy with urinary diversion and pubic bone debridement/resection [5]. Although increasingly recognized in men, this phenomenon is rare in women and thus has not been previously described. This study presents the first characterization of USF in women.

Laroche *et al.* [7] first described USF and consequent pubosymphyseal inflammation in 1995. Additional case reports sporadically brought attention to this devastating condition over the ensuing decades [7–10]. However, it was not until 2012 that USF was characterized in a larger series of patients by Matsushita *et al.* who revealed previous radiotherapy and endoscopic treatment of bladder neck contracture to be common underlying factors [4]. Subsequent, larger series have verified this and identified additional features including congruence of pre-operative urine and intra-operative bone cultures, and histologic accuracy of radiologically suspected pubic osteomyelitis [11, 12].

Similar to studies in men, we observed previous pelvic radiotherapy to be a shared feature in USF formation in two of four women [1, 4]. However, in contrast to these studies, history of prior endoscopic manipulation did not appear to be as imperative for USF formation as only one of these two women underwent previous EBOP. Simply having a chronically indwelling urethral Foley also appears noteworthy as the remaining two women developed USF after having catheters for upwards of 1 year. Given the known risks of urethral erosion associated with long-standing indwelling urethral catheters in women, it is possible that urethral erosion may have played a factor [13].

In 2003, Stern and Clemens presented the first and only described case of pubic osteomyelitis associated with a chronically indwelling catheter in a woman [13]. They detail care of a 40-year-old woman with T10 paraplegia and neurogenic bladder managed with a chronically indwelling urethral catheter for 8 years who presented with unexplained fevers and a sacral wound. Pubosymphyseal cortical destruction was found on CT which interestingly also revealed an anterior pubic fluid collection that was serosanguinous and culture-negative upon aspiration. She was managed with open pubic biopsy and debridement by orthopedists with later plan for urologic reconstruction [13]. Similarly, our present study reflects the known risk of urethral erosion which has been estimated to be up to 50% at 7 years [13, 14]. Furthermore, we suspect that the identified fluid collection may have actually been urine, thereby highlighting the challenge in timely and adequate diagnosis of USF.

While erosion from chronically indwelling urethral catheters may represent a mechanism for USF and its sequelae, prior radiotherapy may also represent another such risk factor. Wignall *et al.* [15] described seven patients, including four women, with a history of pelvic radiotherapy for bladder/cervical cancer who developed pubic osteomyelitis 6–19 years later. Among the women, three were found intra-operatively to have a fistula: two pubovaginal and one puborectovaginal [15]. Surprisingly, none were discovered to involve the urinary tract. The authors concluded an intestinal origin of the proposed infectious etiology of pubic osteomyelitis observed in this setting [15]. However, our study clearly demonstrates USF to be another potential source.

As in men, severe pelvic pain is a common presenting symptom which is tremendously improved or even resolved following extirpative intervention. Similar to Lavien *et al.* [16], we found that the median pain scores improved from 7.5 (IQR 5.5) to 0 (IQR 4) (Table 2). Additionally, one of two women in our study no longer required chronic opioid use post-operatively.

Consideration should be given to urinary catheter diversion, including suprapubic cystostomy tube, when possible as an initial temporizing measure in the acute setting. This may have the additional potential benefit as a long-term management option as observed in carefully selected male populations. However, we should note that this is not always practical in certain situations such as diminutive bladder capacity and severe radiation cystitis (due to intolerable spasm pain and hematuria with clots). Unfortunately, suprapubic catheter placement was only feasible in one of four women and she ultimately required extirpation due to persistent symptoms.

In conclusion, we believe that our study is a call to attention to this rare condition in order to offer potential guidance in its management. Equitable characterization of USF in women may facilitate increased recognition and timely management.

## CONFLICT OF INTEREST STATEMENT

None declared.

## References

[1] Bugeja S, Andrich DE, Mundy AR. Fistulation into the pubic symphysis after treatment of prostate cancer: an important and surgically correctable complication. J Urol 2016;195:391–8. 26301787 10.1016/j.juro.2015.08.074

[2] Shapiro DD, Goodspeed DC, Bushman W. Urosymphyseal fistulas resulting from endoscopic treatment of radiation-induced posterior urethral strictures. Urology 2018;114:207–11. 29305945 10.1016/j.urology.2017.12.020

[3] Osterberg EC, Vanni AJ, Gaither TW, Awad MA, Broghammer JA, Pate SC, et al. Radiation-induced complex anterior urinary fistulation for prostate cancer: a retrospective multicenter study from the Trauma and Urologic Reconstruction Network of Surgeons (TURNS). World J Urol 2017;35:1037–43. 27928592 10.1007/s00345-016-1983-3

[4] Matsushita K, Ginsburg L, Mian BM, De E, Chughtai BI, Bernstein M, et al. Pubovesical fistula: a rare complication after treatment of prostate cancer. Urology 2012;80:446–51. 22698471 10.1016/j.urology.2012.04.036

[5] Andrews JR, Hebert KJ, Boswell TC, Avant RA, Boonipatt T, Kreutz-Rodrigues L, et al. Pubectomy and urinary reconstruction provides definitive treatment of urosymphyseal fistula following prostate cancer treatment. BJU Int Published online January 5, 2021. 10.1111/bju.15333 33403768

[6] Gupta S, Zura RD, Hendershot EF, Peterson AC. Pubic symphysis osteomyelitis in the prostate cancer survivor: clinical presentation, evaluation, and management. Urology 2015;85:684–90. 25733290 10.1016/j.urology.2014.11.020

[7] Laroche F, Perrot S, Menkes CJ. Pubic symphysitis secondary to fistula. Physiopathological hypothesis. Presse Med 1995;24:939–40. 7638145

[8] Kats E, Venema PL, Kropman RF, Kieft GJ. Diagnosis and treatment of osteitis pubis caused by a prostate-symphysis fistula: a rare complication after transurethral resection of the prostate. Br J Urol 1998;81:927–8. 10.1046/j.1464-410x.1998.00606.x 9666790

[9] Kats E, Venema PL, Kropman RF. A rare complication after endoscopic resection of the prostate: osteitis pubis due to a prostate-symphysis fistula. J Urol 1997;157:624. 8996375

[10] Gillitzer R, Melchior SW, Jones J, Fichtner J, Thüroff JW, Thüroff T. Prostatosymphyseal fistula after transurethral resection of the prostate. J Urol 2001;166:1001–2. 11490276

[11] Kahokehr AA, Boysen WR, Schild MH, Nosé BD, Huang J, Eward W, et al. Urinary pubic symphysis fistula leads to histopathologic osteomyelitis in prostate cancer survivors. Urology 2021;148:297–301. 32763316 10.1016/j.urology.2020.07.038

[12] Nosé BD, Boysen WR, Kahokehr AA, Inouye BM, Eward WC, Hendershot EF, et al. Extirpative cultures reveal infectious pubic bone osteomyelitis in prostate cancer survivors with urinary-pubic symphysis fistulae (UPF). Urology 2020;142:221–5. 32389815 10.1016/j.urology.2020.04.095

[13] Stern JA, Clemens JQ. Osteomyelitis of the pubis: a complication of a chronic indwelling catheter. Urology 2003;61:462. 10.1016/s0090-4295(02)02140-4 12597978

[14] McGuire EJ, Savastano J. Comparative urological outcome in women with spinal cord injury. J Urol 1986;135:730–1. 3959193 10.1016/s0022-5347(17)45833-2

[15] Wignall TA, Carrington BM, Logue JP. Post-radiotherapy osteomyelitis of the symphysis pubis: computed tomographic features. Clin Radiol 1998;53:126–30. 10.1016/s0009-9260(98)80059-7 9502089

[16] Lavien G, Chery G, Zaid UB, Peterson AC. Pubic bone resection provides objective pain control in the prostate cancer survivor with pubic bone osteomyelitis with an associated urinary tract to pubic symphysis fistula. Urology 2017;100:234–9. 27591809 10.1016/j.urology.2016.08.035

